# Derivation of Naïve Human Embryonic Stem Cells Using a CHK1 Inhibitor

**DOI:** 10.1007/s12015-023-10613-2

**Published:** 2023-09-13

**Authors:** Carol B. Ware, Erica C. Jonlin, Donovan J. Anderson, Christopher Cavanaugh, Jennifer Hesson, Sonia Sidhu, Savannah Cook, Guillermo Villagomez-Olea, Marshall S. Horwitz, Yuliang Wang, Julie Mathieu

**Affiliations:** 1 Department of Comparative Medicine, University of Washington, Seattle, WA 98195 USA; 2 Institute for Stem Cell and Regenerative Medicine, University of Washington, Seattle, WA 98109 USA; 3 Department of Laboratory Medicine and Pathology, University of Washington, Seattle, WA 98195 USA; 4 Laboratory of Tissue Engineering and Regenerative Medicine, Facultad de Odontología, Universidad Nacional Autónoma de México, 04510 Mexico City, Mexico; 5 Department of Computer Science and Engineering, University of Washington, Seattle, WA 98195 USA

**Keywords:** hESC, CHK1 inhibitor, Naïve, Primed, PICMI, Epigenetic

## Abstract

Embryonic development is a continuum *in vivo*. Transcriptional analysis can separate established human embryonic stem cells (hESC) into at least four distinct developmental pluripotent stages, two naïve and two primed, early and late relative to the intact epiblast. In this study we primarily show that exposure of frozen human blastocysts to an inhibitor of checkpoint kinase 1 (CHK1) upon thaw greatly enhances establishment of karyotypically normal late naïve hESC cultures. These late naïve cells are plastic and can be toggled back to early naïve and forward to early primed pluripotent stages. The early primed cells are transcriptionally equivalent to the post inner cell mass intermediate (PICMI) stage seen one day following transfer of human blastocysts into *in vitro* culture and are stable at an earlier stage than conventional primed hESC.

**Graphical Abstract:**

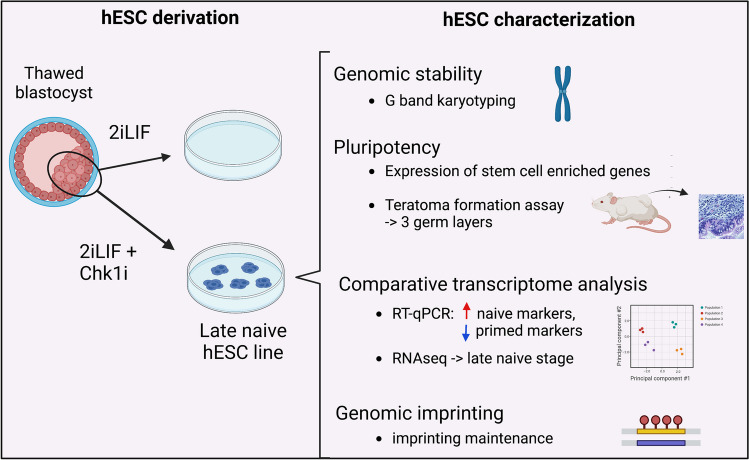

**Supplementary Information:**

The online version contains supplementary material available at 10.1007/s12015-023-10613-2.

## Introduction

The stages of inner cell mass (ICM)/epiblast development are defined by the overarching term “pluripotent”, which means that the cells are capable of forming all three embryonic germ lineages in the developing embryo, to give rise to the individual. Pluripotency in mouse and human cells exists on a continuum of developmental stages [1–4]. Mouse embryonic stem cells (mESC) are considered to be in the naïve state [5, 6], thought to be closely similar to the pre-implantation embryonic ICM. Human embryonic stem cells (hESC) were established in a state more closely similar to the peri- or early post-implantation epiblast in a “primed” state termed EpiSC. If mouse embryos are cultured using hESC culture conditions, they form EpiSC, which are the mouse equivalent of primed hESC [5, 6]. Thus, the mouse naïve state is thought to be earlier in development prior to poising for active differentiation, while the primed state is considered to be poised for active differentiation, prior to gastrulation.

Many studies have described a naïve state that can be achieved in hESC (for reviews see [7–9]). Naïve cells can be obtained through culture conditions altered from conventional primed to toggle the cells back in development from the primed state. Naïve hESC lines have also been directly derived from the ICM of blastocysts [10–14] RNA expression analysis of the naïve stages can further subdivide this developmental state into early (ground state) vs. late naïve states [2–4].

hESC that are reverse toggled from primed to early naïve by altering the culture conditions cannot re-establish normal parental-specific mono-allelic imprinting of several genes upon differentiation, indicating an irreversible loss of the primary imprint [15]. This does not prove to be a problem using the 6iLTF [10] culture conditions, which result in the late naïve on the developmental continuum (see below). Imprint loss has developmental consequences [16] that could seriously impact the utility of a line in future clinical applications. Additionally, there have been excessive karyotype fragility problems associated with lines cultured using the early naïve/ground state culture conditions [17].

Establishment of naïve hESC directly from frozen-thawed human blastocysts has proven inefficient in our hands likely due to cell death upon first passage. Prevention of apoptosis is important to derivation of mouse ESC [18]. The human naïve line we had previously generated, Elf1 [12], was derived from a frozen 8-cell embryo, while frozen blastocysts proved ineffective at generating new hESC lines under the same culture conditions. In this study we explored factors to protect cultured blastocyst ICM from apparent die-off upon naïve hESC derivation using a CHK1 inhibitor for cell line derivation and the subsequent consequences of inhibitors on karyotype and imprint stability.

## Methods

### Derivation and Culture of hESC Lines

Human blastocysts were produced by *in vitro* fertilization for reproductive purposes. Excess were frozen at fertility clinics unafilliated with the University of Washington. Frozen human blastocysts were thawed using a Sage Vitrification Warming Kit (Sage) and placed in Blastocyst medium (Sage) in a 5% CO_2_, 5% O_2,_ 90% N_2_ atmosphere throughout. When the blastocysts had re-expanded 1–4 days following thaw, the zona pellucida was removed mechanically via aspiration through a 100 μm glass capillary and the zona free embryo plated into one well of a 96-well plate seeded with γ-irradiated (3000R) mouse embryonic fibroblast feeders (MEF from CF-1 mice, strain code 023, Charles River, Wilmington, MA). This was denoted as passage 0. Passage 1 was also mechanical using a 100 μm capillary to isolate and breakup the ICM away from the trophoblast. This first passage culture of the *in vitro* inner cell mass used the media described below in the Results and in Supplemental Table 1. Subsequent passages used Accutase (Corning). hESC appeared stably established by passage 4. Henceforth, the cells were passaged two times per week using Accutase and bulk frozen when in hESC culture medium supplemented with a further 10% KOSR using the cryoprotectant dimethylsufloxide (10%, Sigma) using a slow controlled rate freezing method which incorporates an ice crystal seed [19].

All hESC media used are summarized in Supplemental Table 1. Additionally, ROCK1 inhibitor (ROCKi) was added to mTeSR1 (StemCell Technologies) overnight upon passage of primed cells and removed the following day. Cells were toggled backward and forward in development by placing in the appropriate medium directly upon passage. All primed cultures were on Matrigel and all naïve cultures were on mouse embryonic fibroblast (MEF) feeders.

Cells for RT-PCR analysis were grown off of feeders. To accomplish this, naïve cells required the use of a cell-free MEF matrix with the use of a "ghosting" lysis mix (PBS containing 0.5% SDS, 5 mM NH_4_OH; [20]). MEF conditioned medium to supplement the matrix was made using the medium for subsequent hESC culture filtered after culture on irradiated MEF.

### Generation of Teratomas

1 × 10^6^ cells were resuspended in Matrigel supplemented with a cocktail of prosurvival factors [21] and injected into the femoral muscle of SCID-Beige mice (Charles River, Wilmington, MA). Mice were kept under BioSafety containment Level 2. Palpable tumor masses developed in approximately 5 weeks. The tumor bearing mice were sacrificed and tumors were harvested at day 69 (Elm2), day 70 (Elf3) and day 52 (Elf4). Tumor tissues were fixed in 10% formalin (Richard-Allan Scientific) for 24 h, and stored in 70% ethanol until paraffin embedding. Five micrometer sections of the tumors were stained with hematoxylin and eosin using standard protocols. Sections were assessed by a boarded veterinary pathologist.

### High Throughput Oncology Drug Screen

Compound sensitivity profiles of Elf1 naïve (2iLIF) and Elf1 primed (mTeSR) cells were extracted from a high throughput screen of 160 approved and investigational oncology drugs (OncoBlau panel; High Throughput Quellos Core, Institute for Stem Cell and Regenerative Medicine, University of Washington). For naïve cells, 384-well plates were coated with Matrigel (Corning) and kept at room temperature at least 15 min prior to seeding with an irradiated MEF feeder. After overnight incubation at 37 °C, 5% CO_2_, media was aspirated, wells washed once with 50µL PBS and 50µL of ghosting mix was added to the wells. After incubation at room temperature, ghosting detergent was aspirated and wells washed twice with PBS. Primed cells were directly seeded onto Matrigel coated wells. Once prepared in this manner, Elf1 (naïve and primed) were seeded into the wells as a cell suspension (50µL, 2000 and 4000 cells per well). After 24 h incubation at 37 °C, 5% CO_2_, the media containing ROCKi were replaced with fresh media without ROCKi. Immediately following the media exchange, compounds (50nL of AZD7762, SB218078, BML227 or C3742) were added at concentrations ranging from 5 pM to 100 μM and incubated at 37^0^C, 5% CO_2_ for 72 h and viability was assessed using CellTiter Glo (Promega) following a 20 min incubation with quantitation of luminescence derived from intracellular ATP. Resulting dose curves were fitted to a 4 Parameter Logistic Dose Response Model using IDBS XLFit software. Percentage cell viability was reported as relative to the DMSO solvent control. IC_50_ values were calculated by fitting data using a least squares method to the standard four-parameter logistic model where: “Y” = (“Ymin” + (“Ymax”/(1 + ((“X”/”IC50″)^Slope^), and Y = % viability, Ymin = minimal % viability, Ymax = maximal % viability, X = compound concentration, IC50 = concentration of compound exhibiting 50% inhibition of cellular viability, Slope = the slope of the resultant curve. Curve fitting was performed using IDBS XLFit software add-in for Microsoft Excel. Subsequent analysis was performed using Tibco’s Spotfire software.

### Quantitative RT-PCR

RNA was extracted using a Trizol (Life Technologies) or Monarch RNA mini-prep kit (NEB) according to manufacturer’s instructions. RNA samples were treated with Turbo DNase (ThermoFischer) and quantified using Nanodrop ND-1000 (Thermo Scientific). Reverse transcription was performed using an iScript cDNA synthesis kit (BioRad). 10 ng of cDNA was used to perform qRT-PCR using SYBR Green (Applied Biosystems). Primers used are listed in Supplemental Table 2. Real-time RT-PCR analysis was performed on a 7300 real time PCR system (Applied Biosystems). ß-actin was used as an endogenous control.

### Alkaline Phosphatase (AP) Staining

Elf1 2iLIF cells were cultured on irradiated MEF and treated with or without AZD7762 (0.5 μM) for either 24 h or 48 h prior assessement of alkaline phosphatase activity. The cells were fixed in 70% ethanol for 10 min. After 2 washes with PBS 1X, cells were stained using Vector Black substrat Alkaline Phosphatase detection kit (Cat#SK-5200, Vector Laboratories) according to manufacturer's instructions. The colonies were examined visually for appearance of black coloration and pictures were taken using Axio Observer Zeiss microscope.

### H19 Imprinting

Genomic DNA was extracted using QuickExtract (Lucigen) according to the manufacturer’s protocol. RNA was extracted using TRIzol, treated with Turbo DNase and cDNA was generated using iScript cDNA synthesis kit. PCR was performed on gDNA and cDNA using the Phusion Flash High-Fidelity DNA polymerase (NEB). The sequences of the primers used to amplify the region containing a SNP in lncRNA H19 in Elf1 cells were Fwd: GGAACCAGACCTCATCAGCC and Rev: CTGAGACTCAAGGCCGTCTC. The PCR products were run on an agarose gel. The remaining PCR products were purified using ExoSAP-IT PCR clean up reagent (ThermoFisher) and Sanger sequencing was performed by Genewiz.

### RNAseq of Elf3 and Elf4 Cells

RNA-seq libraries were performed using ScriptSeq v2 RNA-Seq Library Preparation Kit (Epicenter). Libraries were sequenced on NextSeq500 in single-end 75-cycle runs. All experiments were performed in duplicate.

### Principal Component Analysis (PCA)

**Fig. 1:**
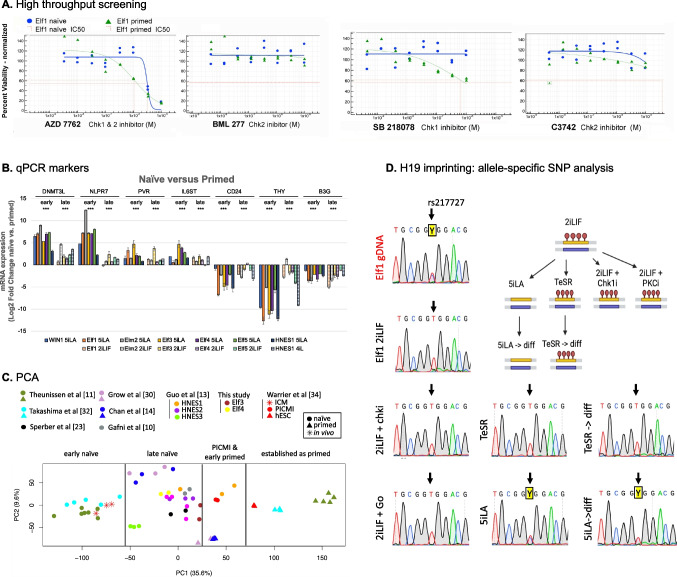
Analysis of new hESC lines generated with CHK1 inhibition, **A**. Analysis of CHK inhibitors for survival of Elf1 naïve vs. primed cells. Viability following exposure to the CHK inhibitors was assessed by luminescence using CellTiter Glo for ATP content. **B**. Expression of naïve and primed markers. qRT-PCR was performed for naïve markers (DNMT3L, NLRP7, PVR and IL6ST) and primed markers (CD24, THY7, BG3) derived from published data [31, 32]. Expression of these genes is shown in newly generated hESC lines (Elm2, Elf3, Elf4 and Elf5), WIN1 [11], Elf1 [12] and HNES1 [13] cultured in ground naïve (5iLA) or late naïve (2iLIF; 4iL), and compared to primed TeSR. The fold changes of expression of markers in naïve conditions (ground or late) versus primed conditions is indicated for each cell line. Error bars indicate the SEM of 3 independent replicates. *** < 0.001. Two-way ANOVA was used to assess whether there are significance differences in means due to the culture conditons (i.e. mean of early naïve vs. mean of late primed and mean of late naïve vs. mean of late primed). **C**. Principal component analysis of RNA-seq from various studies [10, 11, 13, 14, 23, 30, 32, 34] and Elf3 and Elf4 (this study) reveal 4 main pluripotency stages. Batch effect adjustment using ComBat-seq was applied on the combined RNA-seq dataset. **D**. Cells cultured in 2iLIF ± PKCi or CHKi maintain the H19 imprint while 5iLA cells irreversibly erase the imprint as seen upon differentiation. Sanger sequencing traces of Elf1 genomic DNA for H19 containing allele-specific SNP (rs217727, indicated by an arrow) and H19 cDNAs of Elf1 cells cultured in various conditions: 5iLA, 2iLIF, 2iLIF + PKCi, 2iLIF + CHKi, T and cells pushed to undirected differentiation from 5iLA or T. Y = pyrimidine (either C or T nuclotide). Graphical summary of the data was generated using BioRender

Count matrices of RNA-seq samples from this study and others in Fig. 1C were downloaded from Gene Expression Omnibus [22], and the procedure was adapted from [23]. Raw read counts were converted into RPKM (Reads Per Kilobase of transcript, per Million mapped reads) values. The sum of RPKM values for each gene is calculated across all samples, and genes with a total less than 3 RPKM were filtered out. RPKM matrix was transformed to log2, and protein-coding genes were selected for further analysis. Because naïve and primed samples came from different labs, the sva package [24] was used to correct for batch effects. The expression matrix was centered. Principal component analysis was done using the *prcomp* function in R on filtered, batch corrected and centered expression matrix.

### Whole Exome Sequencing

Genomic DNA was obtained from approximately one million Elf1 cells. Genomic DNA was enriched for exomic regions and sequencing libraries prepared using the Agilent SureSelect All Exon Target Enrichment System (Human All Exon Kit V5). Libraries were analyzed on an Illumina HiSeq 2000 system with 100 bp forward and reverse reads resulting in 7.6 billion reads mapped, on average, for both samples. Reads were aligned to hg19 with BWA (v0.7.7) and variants identified using the UnifiedGenotyper tool contained in the Genome Analysis Toolkit (GATK 3.1) with default settings for DNA analysis. We obtained an average targeted region fold coverage of 96 for both samples.

### Karyotype Analysis

Karyotype was analyzed via G-banding at Diagnostic Cytogenetics, Seattle, WA. To explore a possible interaction of CHK1i with colcemid appearing as tetraploids, karyotype analysis also proceeded without the use of colcemid (with reduced efficiency).

### Statistical Analysis

All experiments were performed at least in triplicate. Data are expressed and plotted as the mean ± SEM as indicated in the figure legend. Statistical analysis was performed using GraphPad Prism statistics software. Two-way ANOVA was applied to test for significant differences in means between groups/conditions. A p value of less than 0.001 was considered extremely significant and indicated as ***.

## Results

### Definitions of Culture Conditions That Impact Human Pluripotent Cell State

In this study, we used published hESC culture conditions summarized in Supplemental Table 1. For early naïve culture we used **5iLA** conditions (MEK inhibitor, GSK3 inhibitor, ROCK inhibitor, BRAF inhibitor and SRC inhibitor with human leukemia inhibitor factor (hLIF) and Activin A; [11]). For human late naïve culture we used either **4iL** (MEK inhibitor, GSK3 inhibitor, ROCK inhibitor and PKC inhibitor with hLIF [13]); **2iLIF** (MEK inhibitor and GSK3 inhibitor + hLIF, IGF1 and bFGF; [12]), or **6iLTF** conditions (MEK inhibitor, GSK3 inhibitor JNK inhibitor, P38 inhibitor, PKC inhibitor and ROCK inhibitor + hLIF, TGFβ and bFGF; [10]). For human primed culture all cell lines were grown in mTeSR (**T**) medium without added inhibitors or additional growth factors.

Prior to establishing new hESC lines, we used existing lines established directly as naïve: Win1 [11], Elf1 [12] and HNES1 [13]. Elf1 was derived from a frozen 8-cell embryo that was further cultured upon thaw to blastocyst, while the cryopreservation stages of the embryos that generated Win1 and HNES1 were not directly stated. Embryos frozen as 8-cell are rarely donated by patients who have unneeded frozen embryos following clinical fertility treatment because clinics have moved to storage of blastocysts for direct uterine transfer at thaw. Because of the availability of frozen blastocyst stage human embryos, we aimed to duplicate the conditions that resulted in Elf1 using a medium that contained 2iLIF plus factor(s) that would allow the cultures to flourish.

### Cell Death in Naïve and Primed hESC

Human pluripotent cells actively use the Fanconi Anemia (FA) pathway to facilitate DNA repair [25–27]. A recent publication indicated that an inhibitor of CHK1 (CHK1i; AZD7762) allowed FA patient fibroblasts to reprogram to iPSC by attenuating cell death [28]. Notably, they did not detect karyotype fragility although predicted by CHK1 loss in the context of FA mutation. We used a screen for survival of naïve vs. primed Elf1 cells which included one CHK1 and 2 inhibitor (AZD7762), one CHK1 only inhibitor (SB218078) and two CHK2 only inhibitors (BML277 and C3742) (Fig. 1A). The naïve cells tolerated higher levels of CHK1 inhibition compared to primed cells, which indicated that naïve cells were tolerant and survive with reduced CHK1 activity relative to primed cells. There was no difference in the presence of CHK2 inhibition between naïve and primed in that neither stage of pluripotency appeared to be influenced by the loss of CHK2 activity at the levels of inhibitors used. Thus, AZD7762, as used in generating FA patient iPSC cells [28], was used to assist in establishment of naïve hESC. We first showed that naïve cells treated with AZD7762 were still positive for Alkaline Phosphate staining (Supplemental Fig. 1A).

### Establishment of Naïve hESC From Frozen Blastocysts

**Fig. 2:**
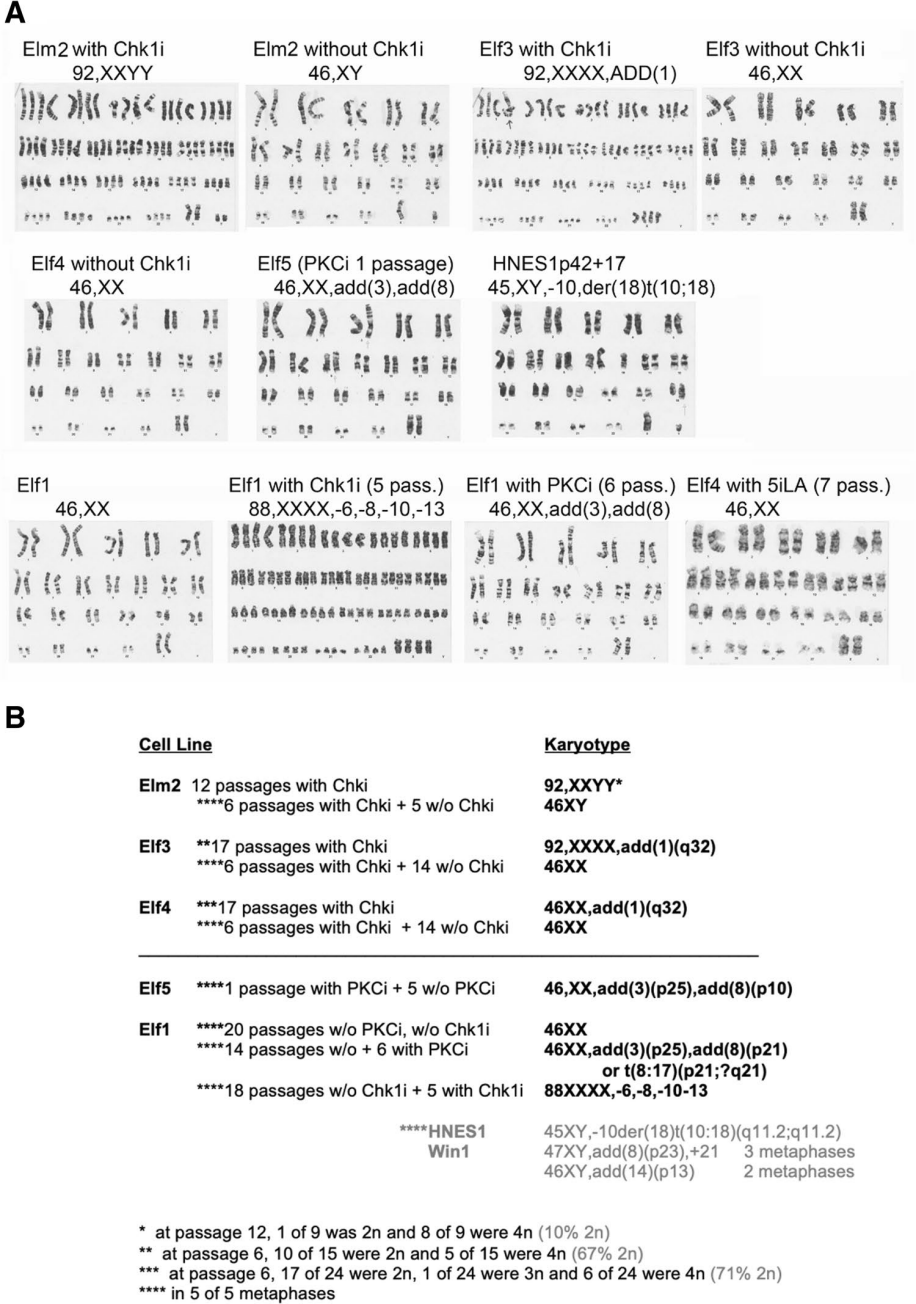
G-banded karyotype analysis of newly generated hESC lines, **A**. G-banded karyotype analysis was performed in naïve hESC (Elf1, Elm2, Elf3, Elf4, Elf5 cultured in 2iLIF and HNES1 cultured in 4iL) Elf5 (PKCi 1passage were established in 4iL and transferred to 2iLIF upon the second passage). Denotations of HNES1 passage number follows the convention of labeling from the Nichols lab from which they were obtained with a passage number change up from the second number upon each passage in 4iL. Elf1 number of passages in inhibitors is denoted as the number of passages in the particular inhibitor, e.g. “Elf1 with CHK1i (5 pass.) indicates Elf1 with a normal karyotype subsequently passaged 5 times in the presence of CHK1i. The final analysis of karyotype is printed below the cell line details. **B**. Table showing ploidy effects of culture in CHK1i or PKCi. Naïve hESC Elf1 [12], Elm2, Elf3, Elf4 and Elf5 were cultured in 2iLIF media supplemented with 0.5 mM CHK1i, 1 mM PKCi or 50 mM ZVAD for the indicated number of passages prior to G-banded karyotype analysis. Five metaphases for each culture were assessed

Human frozen blastocysts were thawed and cultured in 2iLIF without a further additive [12], or in 2iLIF with CHK1i (0.5 μM AZD7762) or protein kinase C inhibitor (PKCi, 1 μM Gö6983 in published medium as used in [13]) or in 2iLIF plus Z-VAD-FMK (ZVAD, 50 μM, as an alternate means of reducing apoptosis through caspase inhibition). Embryos that were thawed and cultured in 2iLIF with no further inhibitor additions infrequently survived beyond the second passage, where isolation of the ICM away from the trophoblast of the adherent embryo was considered passage one. These isolates appeared to arrest and die, although less frequently, morphological differentiation also occurred. Presence of CHK1i and PKCi allowed direct and relatively efficient derivation (3 lines from 12 embryos that survived freeze/thaw and 1 line from 14 that survived freeze/thaw, respectively) to establish naïve hESC lines capable of stable long-term culture (Table 1). Caspase inhibition by ZVAD appeared to have no obvious impact upon survival (no lines from 20 embryos that survived freeze/thaw). Because pluripotent cells cultured with PKCi (HNES1) indicated that it induced karyotype fragility (Fig. 2) the one line derived under these conditions was switched directly to 2iLIF after ICM isolation between passage 1 and passage 2 to minimize exposure to PKCi.

**Table 1:** Effect of CHK1 and PKC inhibitors on establishment of new hESC lines. Indicated are the number of embryos obtained from fertility clinics cryopreserved at various developmental stages, how many survived the thaw and were then able to generate new hESC lines in 2iLIF media with or without inhibitors (CHK1i, PKCi, ZVAD). All the embryos cultured with the inhibitors had been frozen at the blastocyst stage

Stage	# Thawed	# Survived	Cell lines
1-cell	77	31	0
2-cell	12	2	0
4–8 cell	39	14	1
Blastocyst:	566	366	0
w/ CHK1i	20	12	3
w/ PKCi	22	14	1
w/ ZVAD	35	20	0

The new lines were named "El" for the Ellison Stem Cell Core, where culture took place, and either "m" or "f" for male or female. Lines Elm2, Elf3 and Elf4 were derived in the presence of CHK1i and Elf5 was derived in PKCi (4iL). Karyotypes were assessed on the four newly derived lines (Fig. 2A). If CHK1i is present throughout culture, the cells can move toward tetraploidy, but if CHK1i is removed these cultures revert to normal 2n karyotypes (Fig. 2B). In addition, known 2n Elf1 were cultured in CHK1i and these cells, too, moved to a tetraploid karyotype relative to cells cultured side-by-side without CHK1i. 2n Elf1 were also cultured in 2iLIF plus PKCi for 6 passages alongside cultures without PKCi. All metaphases analyzed had developed an abnormal karyotype if PKCi was present, but remained normal without PKCi.

To determine if tetraploidy was real or an artefact from the G-banding karyotype process by exposure to colcemid, Elf1 cells cultured with CHK1i for 11 passages were karyotyped. 35/65 cells were diagnosed as tetraploid, while a companion culture was exposed to CHK1i for 12 passages and CHK1i removed 8 h prior to karyotype. Again 34 of 64 cells appeared tetraploid suggesting that it is not the presence of CHK1i during the karyotype process that resulted in an artefactual tetraploid diagnosis, but that cultures exposed to CHK1i gain an extra chromosome complement over time in culture, which can be resolved to diploid upon further culture without CHK1i, likely due to a survival advantage of 2n cells. Also, though difficult to find good spreads, absence of colcemid in the process did not affect the number of 4n cells detected.

### Diagnostic Expression Differences Between Naïve and Primed hESC States

hESC lines established in CHK1i were compared with other previously reported lines to determine integrity and utility of these hESC lines. They expressed factors identifying the undifferentiated state (OCT4, NANOG and SOX2) (Supplemental Fig. 1B) [29] and were able to generate teratomas when injected into SCID-Beige mice, demonstrating the ability of the newly generated lines to differentiate into the three germ lineages (Supplemental Fig. 1C). We identified several genes that should distinguish naïve vs. primed hESC [30, 31] and ran quantitative reverse transcription PCR (qRT-PCR) analysis to determine levels in the new lines and in those previously reported relative to lines cultured in mTeSR (Fig. 1B). Naïve hESC markers DNMT3L and NLRP7 were expressed at the highest levels in cells cultured in 5iLA early naïve conditions. All naïve cells expressed PVR and IL6ST relative to primed. Conversely, 5iLA cells had the lowest expression of primed markers CD24 and THY. All naïve cells had reduced expression of B3G.

### Principal Component Analysis of Developmental Stage

PCA re-analysis of RNA expression of reported data places hESC within a developmental continuum when compared to the ICM. PCA puts both 5iLA [11] and cells exposed to short term NANOG and KLF2 overexpression [32] close to the embryonic ICM, while 2iLIF [12], 3iL [14], 4iL [13], 6iLTF [10] cells are developmentally closer to, but not yet, primed (Fig. 1C). There is an indication that from this stage they retain the capacity to form the germ lineage [33]. Analysis of CHK1i-generated hESC Elf3 and Elf4 are found clustering with other late naïve hESC (Fig. 1C). Of note, cells established as early naïve move to late primed when placed in primed culture medium, while late naïve cells only advance to an early primed state. This early primed state equivalent was observed in the study that described PICMI cells, one day following ICM subculture [34]. These PICMI cultures were transitioned into primed medium to establish late primed hESC lines. The PICMI stage was fleeting in this context. Very likely development continues from ICM to the PICMI state within 24 h of disrupting the embryo and then either reverse toggles to naïve or forward toggles to primed, depending on the medium that the PICMI cells experience. Cells derived as late naïve stably progressed to the early primed stage when placed in primed medium. Early primed cells were transcriptionally equivalent to the PICMI.

### H19 Imprint Maintenance

H19 is paternally imprinted so that the maternal allele remains unmethylated in primed cells. Monoallelic imprint has been explored in hESC in primed cells and cells reverse toggled to naïve via culture in 5iLA [17]. Primed cells had an appropriate monoallelic methylation pattern on H19. But when cells were toggled back under 5iLA conditions, the imprint was erased so that both alleles were unmethylated. Differentiation back to primed did not allow appropriate monoallelic imprint re-patterning, but maintained hypo-methylation on both the maternal and paternal H19 alleles. Elf1 contains a SNP (rs#217727, as determined by whole exome sequencing) that allows distinction of methylation of the maternal vs. paternal alleles on H19. Sequencing of Elf1 determined the maternal allele carries a "T" and remains unmethylated and the paternal allele carries a "C" that becomes methylated. This allows diagnosis of parental H19 expression by RT-PCR. Elf1 cultured under different conditions were analyzed for imprint erasure and ability to re-establish imprint on the paternal allele upon development (Fig. 1D). Mono-allelic expression is maintained in Elf1 2iLIF and erased in Elf1 5iLA. Appropriate mono-allelic, uniparental imprint could not be appropriately re-established if Elf1 is moved from 5iLA to T, consistent with the Pastor et al. report [15]. Allelic expression of H19 was also analyzed in Elf1 cultured in 2iLIF with either PKCi or CHK1i. In both instances, H19 imprint was monoallelic. This indicates that moving established *in vitro* hESC cultures back in development to the early naïve state had the side-effect of erasing parental imprint to the extent that parental methylation fidelity could not be re-established in the primed state and presumably by further differentiation. Reversing development to the late naïve, but not the early naïve using either CHK1i or PKCi, allowed parental imprint maintenance. This was also consistent with the Pastor et al. report [15] where they found 6iLTF cells established as late naïve, capable of maintaining parental imprint pattern. Combination of the H19 imprint and PCA results indicated that establishment of a hESC line too early in development can disrupt normal developmental imprint re-establishment as the cells progress in development.

### Stage Transition Ability

The ability of each line to toggle between early and late naïve, and early and late primed was assessed. Moving cells in 5iLA (early naïve) into 2iLIF (late naïve) always failed, while 5iLA cells could be moved directly to late primed and 2iLIF cells could be moved back to 5iLA (early naïve). (Supplemental Table 3 and Supplemental Fig. 2). Also, all lines could be moved from early and late primed back to either 2iLIF or 5iLA without the need of overcoming any blocks. The cause of a block in cells transitioning from early to late naïve is unknown. Possibly cells cannot exist in the late naïve state if the parental imprint fidelity is lost.

## Discussion

The primary findings in this study are that inhibition of CHK1 in human embryos allows efficient, karyotypically normal late naïve hESC derivation from frozen blastocysts. Inhibition of CHK1 is important at the PICMI stage that allows transition from *in vivo* to *in vitro* pluripotent culture. Late naïve cells grown as primed in mTeSR appear not to stabilize as far along in development as conventional primed cell lines, whereas early naïve cells cannot transition to late naïve and early primed stages. Generation of early and late naïve hESC is likely to involve reverse toggling from PICMI [34], while the cells established at late naïve can mimic a stable PICMI state when moved to primed conditions, while early naïve do not. Thus, late naïve and primed cells maintain the developmental integrity of the ICM.

The ease of establishing Elf1 from a frozen 8-cell embryo as opposed to our inability to start new lines from frozen blastocysts was unexpected. Perhaps blastocyst cryopreservation, when the maternal proteins are diminished after zygotic genome activation at the 8-cell stage [35], had an effect. If true, non-cryopreserved blastocysts would presumably not need either CHK1i or PKCi to establish a naïve hESC line. Non-cryopreserved human embryos donated to research are generally unavailable while those cryopreserved at the 1–8 cell stage are less available now that fertility clinics routinely freeze embryos cultured to blastocyst.

Mouse Chk1 null mutation in an early embryo is lethal after the 8-cell stage due to reduced blastocyst survival with loss of the ICM [36, 37]. The assumption is that mouse and human ES cells will die in the face of CHK1i, while previous reports indicate the hiPSC do not [27]. The human difference in this regard is not obvious. Mouse embryogenesis follows a rapid temporal developmental pathway relative to humans with regard to genome activation (at 2-cell for mouse and 8-cell for human) [35]. Also, mouse pluripotent cells represent an earlier developmental timepoint relative to the ICM than do conventional primed hESC [5, 6] with differing culture conditions. Possibly exposure of thawed blastocysts to CHK1i is not directly relateable to a null mutation present throughout early development. Mouse blastocysts would need to be thawed under conditions used in this study to determine how similar they are relative to humans in response to CHK1i.

Tetraploid pluripotent cells survived in culture if CHK1 was inhibited. Causes of tetraploidy during hESC derivation to a late naïve state is open to speculation and would likely hinge on bypass of the G2 checkpoint in combination with bypass of apoptosis [38]. DNA repair in ESC is via microhomology-mediated end joining (MMEJ), normally a highly error-prone but quick process. TP53BP1 provides DNA end protection in this context and pluripotent stem cells exclusively utilize ligase 3 for end-joining [39]. Upon failed repair, pluripotent stem cells can effect unconventional rapid apoptosis via Bax-activated p53 resident on the surface of the Golgi apparatus [40]. Combined, human pluripotent stem cells take advantage of a rapid means of genome protection that is not disrupted by CHK1 inhibition.

Naïve culture conditions invariably require the presence of an inhibitor of MAP kinase signaling. This is often accompanied by an inhibitor of GSK3 to, presumably, enhance WNT signaling [41]. Recently, concerns have been raised over the karyotype integrity of cells exposed to MAP kinase signaling inhibition in long-term culture [42]. In particular, the cells cultured under 5iLA conditions fall victim to karyotypic abnormalities after approximately 10 passages, but remain stable when MEKi exposure is reduced [42]. 2iLIF conditions utilize equivalent MEK inhibition to 5iLA. If aneuploidy does prove to be problematic with regard to maintenance of late naïve pluripotent lines a possible solution would be to maintain lines under early primed culture, while reverse toggling the early primed cultures to a later naïve state when needed.

Use of FGF in late naïve hESC culture was an initial concern. It was thought that naïve cells would be driven to a primed stage through MEK. However, FGF has effects beyond influence on the MEK pathway, especially when a MEK inhibitor is present. Protection during reverse toggle conversion of primed to 5iLA culture conditions was more efficient if FGF was present [43]. It is likely not a requirement of naïve cell culture, but it appears to be protective to improve survival.

In summary, inhibition of CHK1 facilitates establishment of late naïve hESC directly from blastocysts. Human pluripotent cells are able to sustain culture in four distinct developmental stages reflective of embryonic ICM developmental progression adding to our understanding of human early development and the clinical utility of these cells. The late naïve stage appears to reflect the earliest *in vitro* equivalent to the blastocyst for unencumbered development while the early primed stage reflects a stable equivalent to the PICMI, essentially allowing a culture reflecting a close embryonic equivalent.

### Supplementary Information

Below is the link to the electronic supplementary material.

Supplementary file1 Supplemental Figure 1. Pluripotency analysis of newly generated hESC lines. A. Alkaline Phosphatase staining. Elf1 cells cultured in 2iLIF naïve conditions treated with CHK1i (0.5 μM AZD7762) for either 24h or 48h are positive for Alkaline Phosphatase staining. Scale bar=100 µm. B. Expression of factors that define the undifferentiated state (OCT4, NANOG, SOX2) by qRT-PCR in newly generated hESC lines (Elm2, Elf3, Elf4 and Elf5), and Elf1[12] cultured in 2iLIF naïve conditions and normalized to β-actin expression. Error bars indicate the SEM of 3 independent replicates. C. Teratoma analysis. Hematoxylin and eosin stained sections of teratomas obtained after injection of Elm2, Elf3 or Elf4 cells in immunodeficient mice. Differentiated tissues from all three germ layers are apparent. Views of well-differentiated teratomas are shown. NE = neuroectoderm. e = endoderm. Scale bar=100 µm. Supplemental Figure 2. Toggling capacities between pluripotent states, Bright field images of Elf1 after various toggling conditions. Morphology analysis shows that all cells differentiated from 2iLIF conditions flatten into primed colonies (top panels). Panels below indicate cultures in the ground state (5iLA) cannot be pushed to a stable late naïve state in 2iLIF, even if cells are converted to early primed prior to conversion to ground state and are differentiated or gone by passage 3 in 2iLIF. Scale bar=200 µm (PDF 7.16 MB)

Supplementary file2 Supplemental Table 1. Culture media components for naïve hESC culture (PDF 42 KB)

Supplementary file3 Supplemental Table 2. Sequences of RT-qPCR primers used in this study (PDF 22 KB)

Supplementary file4 Supplemental Table 3. hESC line culture differences in ability to toggle between stages (PDF 13.7 KB)

## Data Availability

All data generated or analyzed during this study are included in this published article and its supplementary information files. The HNES1 and WIN1 lines were a kind gift from Drs Nichols and Jaenisch, respectively.

## Code Availability

All code used for analysis is available as referenced.
