# Supplementary figures and images for: Derivation of Naïve Human Embryonic Stem Cells Using a CHK1 Inhibitor
Source: Stem Cell Rev Rep. 2023 Sep 13;19(8):2980–90. doi: 10.1007/s12015-023-10613-2 (PMC10662141; doi:10.1007/s12015-023-10613-2)

Supplemental Fig1

A. Alkaline Phosphatase staining

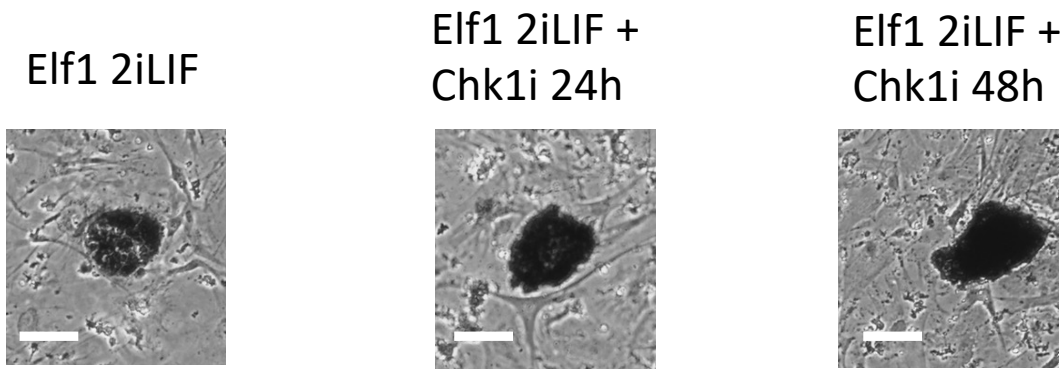

B.

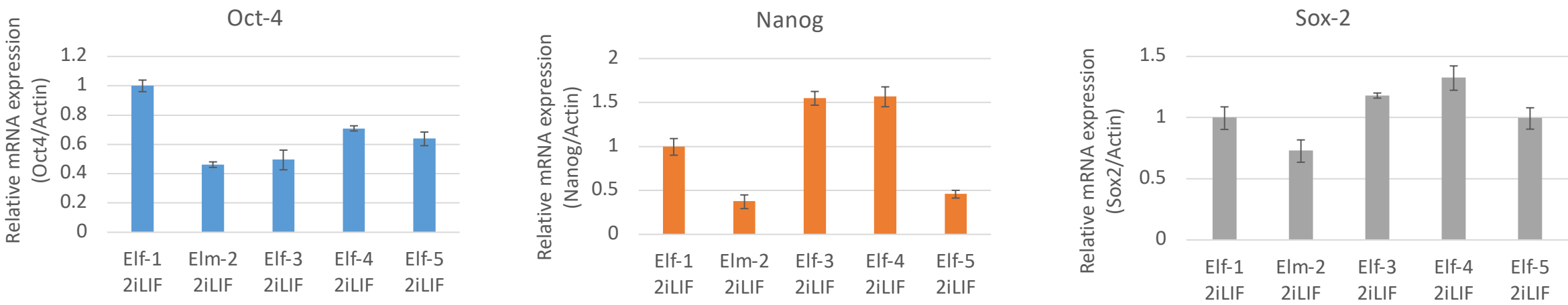

C. Teratoma assay

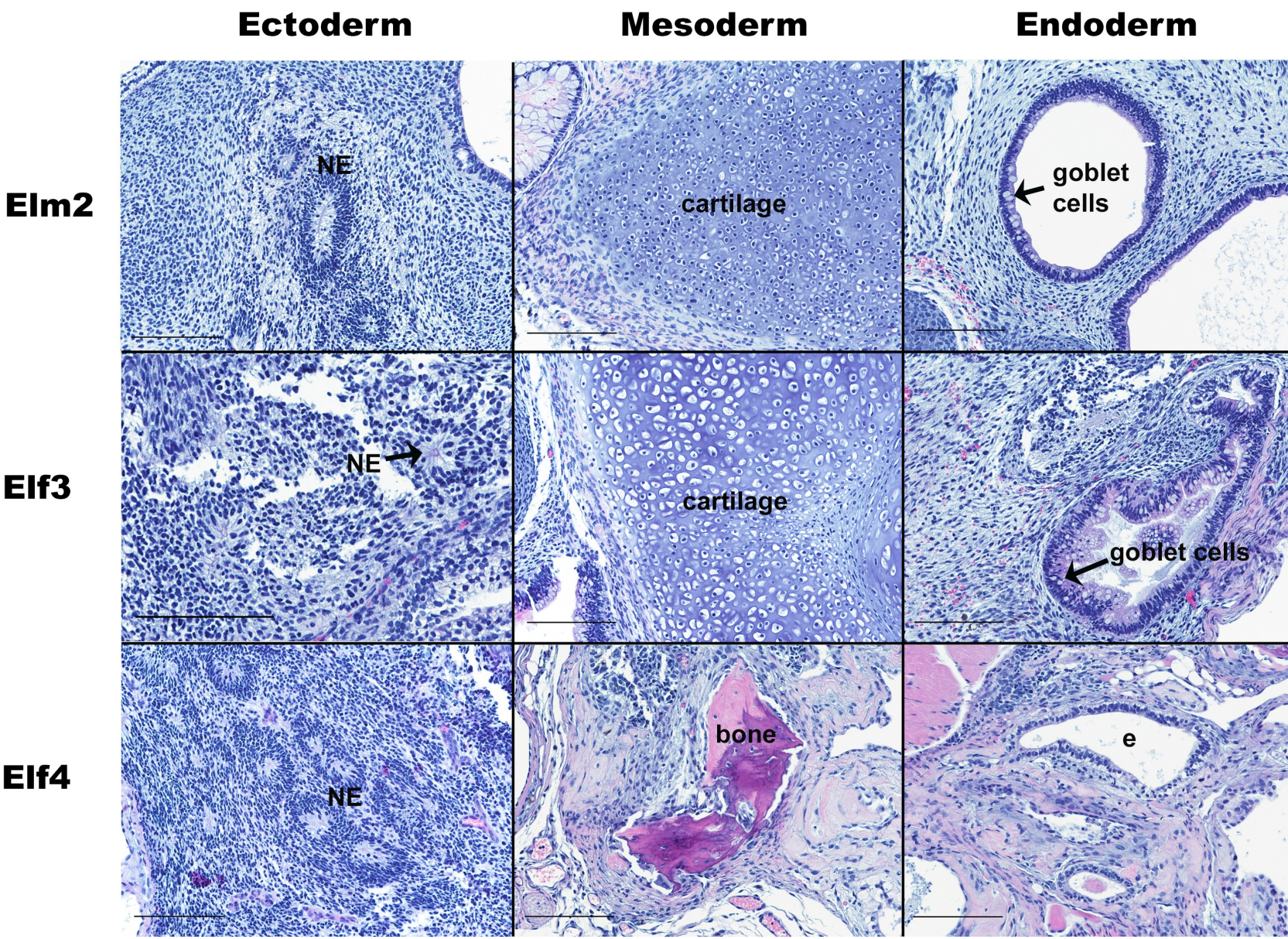

Supplemental Fig2

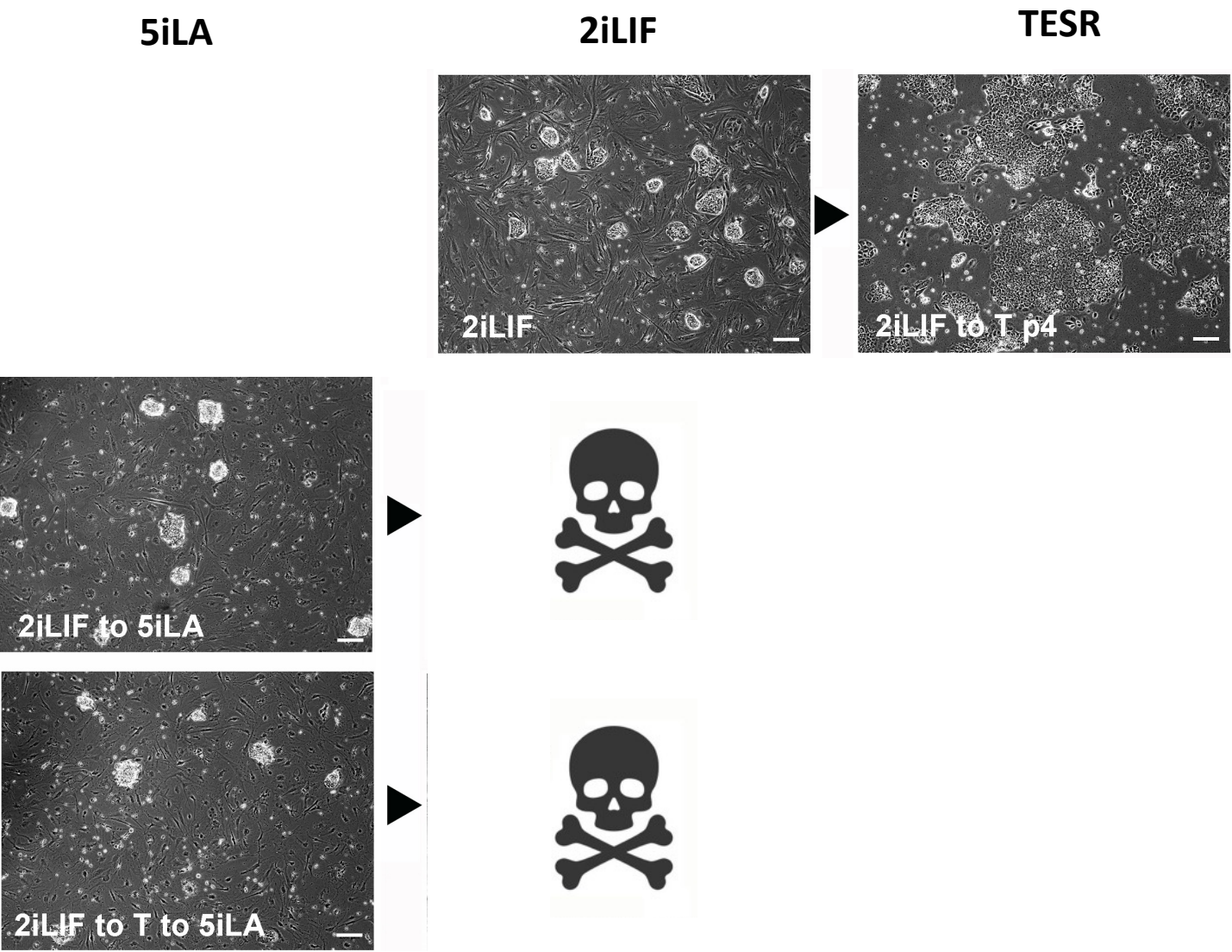

Supplement: Supplementary file 1 — Supplementary file1 Supplemental Figure 1. Pluripotency analysis of newly generated hESC lines. A. Alkaline Phosphatase staining. Elf1 cells cultured in 2iLIF naïve conditions treated with CHK1i (0.5 μM AZD7762) for either 24h or 48h are positive for Alkaline Phosphatase staining. Scale bar=100 µm. B. Expression of factors that define the undifferentiated state (OCT4, NANOG, SOX2) by qRT-PCR in newly generated hESC lines (Elm2, Elf3, Elf4 and Elf5), and Elf1[12] cultured in 2iLIF naïve conditions and normalized to β-actin expression. Error bars indicate the SEM of 3 independent replicates. C. Teratoma analysis. Hematoxylin and eosin stained sections of teratomas obtained after injection of Elm2, Elf3 or Elf4 cells in immunodeficient mice. Differentiated tissues from all three germ layers are apparent. Views of well-differentiated teratomas are shown. NE = neuroectoderm. e = endoderm. Scale bar=100 µm. Supplemental Figure 2. Toggling capacities between pluripotent states, Bright field images of Elf1 after various toggling conditions. Morphology analysis shows that all cells differentiated from 2iLIF conditions flatten into primed colonies (top panels). Panels below indicate cultures in the ground state (5iLA) cannot be pushed to a stable late naïve state in 2iLIF, even if cells are converted to early primed prior to conversion to ground state and are differentiated or gone by passage 3 in 2iLIF. Scale bar=200 µm (PDF 7.16 MB) [file 12015_2023_10613_MOESM1_ESM.pdf]
