# Supplementary material for: Derivation of Naïve Human Embryonic Stem Cells Using a CHK1 Inhibitor
Source: Stem Cell Rev Rep. 2023 Sep 13;19(8):2980–90. doi: 10.1007/s12015-023-10613-2 (PMC10662141; doi:10.1007/s12015-023-10613-2)
Supplement: Supplementary file 2 — Supplementary file2 Supplemental Table 1. Culture media components for naïve hESC culture (PDF 42 KB) [file 12015_2023_10613_MOESM2_ESM.pdf]

**Supplemental Table 1.** Culture media Components for naïve hESC culture				
per 100 ml	**5iLA** Theunissen et al., 2014	**4iL** Guo et al., 2016	**2iLIF** Ware et al., 2014	**6iLTF** Gafni et al., 2013
**Base Media**:				
DMEM/F12 [1]	48 ml	49 ml	80 ml	80 ml
Neurobasal[1]	48 ml	49 ml	-	-
N2[1]	1 ml	1 ml	-	5 ml
B27[1]	2 ml	2 ml	-	-
KOSR[1]	-	-	20 ml	15 ml
non-essential AA (100x)[1]	1 ml	1 ml	1 ml	1 ml
Penicillin/Streptomycin (100x)[1]	1 ml	-	1 ml	-
L-glutamine[1]	1 mM	2 mM	2.5 mM	1 mM
Ascorbic Acid[2]	-	250 µM	-	50 µg/ml
β-mercaptoethanol[2]	0.1 mM	0.1 mM	0.1 mM	0.1 mM
**Growth Factors**:				
hLIF[4]	20 ng/ml	10 ng/ml	10 ng/ml	20 ng/ml
Activin[5]	10 ng/ml	-	-	-
Insulin[1]	-	10 µg/ml	-	12 µg/ml
IGF1[6]	-	-	2 ng/ml	-
FGF2[1]	-	-	10 ng/ml	8 ng/ml
TGFβ[2]	-	-	-	1 ng/ml
**Inhibitors**:				
MEK (PD0325901)[3]	1 µM	1 µM	1 µM	1 µM
GSK3: (CHIR99021)[3]	-	1 µM	1 µM	3 µM
(IM-12)[3]	1 µM	-	-	-
ROCK (Y-27632)[3]	10 µM	10 µM	-	5 µM
B-RAF (SB590885)[3]	0.5 µM	-	-	-
SRC (WH-4-023)[3]	1 µM	-	-	-
PKC (Gö6983)[3]	-	2.5 µM	-	5 µM
JNK (SP600125)[3]	-	-	-	10 µM
p38 (BIRB796)[3]	-	-	-	2 µM

1 Gibco
2 Sigma
3 SelleckChem
4 Speed Biosystems
5 Humanzyme
6 Peprotech
