# Supplementary material for: Derivation of Naïve Human Embryonic Stem Cells Using a CHK1 Inhibitor
Source: Stem Cell Rev Rep. 2023 Sep 13;19(8):2980–90. doi: 10.1007/s12015-023-10613-2 (PMC10662141; doi:10.1007/s12015-023-10613-2)
Supplement: Supplementary file 3 — Supplementary file3 Supplemental Table 2. Sequences of RT-qPCR primers used in this study (PDF 22 KB) [file 12015_2023_10613_MOESM3_ESM.pdf]

Supplemental Table 2

	Gene	Forward	Reverse
Naïve	CD130 (IL6ST)	CTGTATCACAGACTGGCAACAAG	GCATTTGCTCTCTGCTAAGTTCC
	CD155 (PVR)	ACTCAGGCATGTCCCGTAAC	GTCTGTGGATCCTGGGAAGA
	DNMT3L	CTCTCAAGCTCCGTTTCACC	TCGCTGGTTAGCCTTGACTT
	NLRP7	CTTCTGTGCGGATTCTTTGTGA	TTTTTAATCTCCACTTTCTGCAGATG
Primed	CD24	TTTGGGAAGTGAAGACTGGAA	ACTGCAGAATCAAGCCCACT
	CD57 (B3GAT1)	TCCTGAACTTACCGCCACTCA	CGTCTCTTCGGCATCTCCAA
	CD90 (THY1)	ATCTCCTCCCAGAACGTC	ATCTCTGCACTGGAACTTG
	Actin	TCCCTGGAGAAGAGCTACG	GTAGTTTCGTGGATGCCACA
