# Supplementary material for: Derivation of Naïve Human Embryonic Stem Cells Using a CHK1 Inhibitor
Source: Stem Cell Rev Rep. 2023 Sep 13;19(8):2980–90. doi: 10.1007/s12015-023-10613-2 (PMC10662141; doi:10.1007/s12015-023-10613-2)
Supplement: Supplementary file 4 — Supplementary file4 Supplemental Table 3. hESC line culture differences in ability to toggle between stages (PDF 13.7 KB) [file 12015_2023_10613_MOESM4_ESM.pdf]

Supplemental Table 3

Cell line	Initial Medium Additives	Target Medium Additives	# passages to stable culture
Elf1	5iLA	2iL-I-F	Failed
	5iLA	TeSR	2
	2iL-I-F	5iLA	3
	2iL-I-F	T	1
	T	2iL-I-F	1
	T	5iLA	3
Elf4	5iLA	2iL-I-F	Failed
	5iLA	TeSR	1
	2iL-I-F	5iLA	1
	2iL-I-F	T	3
	T	2iL-I-F	1
	T	5iLA	1
Wibr3	5iLA	2iL-I-F	Failed
	5iLA	TeSR	3
	2iL-I-F	5iLA	2
	2iL-I-F	T	1
	T	2iL-I-F	2
	T	5iLA	4
